# Reply to Kenyon, “Are Differences in the Oral Microbiome Due to Ancestry or Socioeconomics?”

**DOI:** 10.1128/mSystems.00891-19

**Published:** 2020-03-10

**Authors:** Yaohua Yang, Wei Zheng, Qiuyin Cai, Martha J. Shrubsole, Zhiheng Pei, Robert Brucker, Mark Steinwandel, Seth R. Bordenstein, Zhigang Li, William J. Blot, Xiao-Ou Shu, Jirong Long

**Affiliations:** a Division of Epidemiology, Department of Medicine, Vanderbilt Epidemiology Center, Vanderbilt-Ingram Cancer Center, Vanderbilt University Medical Center, Nashville, Tennessee, USA; b Department of Pathology, New York University School of Medicine, New York, New York, USA; c Rowland Institute, Harvard University, Cambridge, Massachusetts, USA; d International Epidemiology Field Station, Vanderbilt University Medical Center, Rockville, Maryland, USA; e Department of Biological Sciences, Vanderbilt University, Nashville, Tennessee, USA; f Department of Pathology, Microbiology, and Immunology, Vanderbilt University, Nashville, Tennessee, USA; g Department of Biostatistics, University of Florida, Gainesville, Florida, USA

**Keywords:** host-microbial interaction, oral microbiome

## REPLY

We thank Dr. Kenyon for his careful reading of our paper (1, 2). First, he speculated that the differences in relative abundance of the 13 most common taxa between European-Americans (EAs) and African-Americans (AAs) may be due to inadequate control of covariates. Then, he expressed concerns on the measurement of periodontal health. At last, he conjectured that the higher prevalence of four periodontal bacteria among AAs than EAs may be due to the fact that dental caries was more common in AAs and populations with lower income and poorer education (3–5).

To address his first remark, we investigated the associations between the common taxa and ancestry, stratified by age (40 to 60 years and 60 to 80 years), gender (female and male), annual household income (<$15,000 and ≥$15,000), education (<high school and ≥high school), smoking (current smoker and non-current smoker), and tooth loss (none and any), respectively. Consistent with the statistical methods described in our paper, centered log-ratio (clr) transformation was used to normalize taxon read counts and the associations of ancestry with clr-transformed taxon abundance were evaluated using linear regression analyses. When stratified by one covariate, all other covariates were adjusted in regression models (1). In all these stratified analyses, the majority of the 13 common taxa were associated with ancestry within each stratum (*P* < 0.05) and the association directions are consistent between the two strata for each covariate (Table 1). These results suggest that the differential abundance for these common taxa between AAs and EAs is less likely due to the inadequate adjustment of these covariates. Regarding the oral health measurement, we indeed acknowledged this limitation in the original publication, namely, lacking a comprehensive oral health assessment at the baseline examination during the enrollment (1). Finally, to determine whether the higher prevalence of the four periodontal bacteria among AAs than EAs was affected by socioeconomic differences between the two groups, we conducted association analyses of ancestry with prevalence of these four bacteria, stratified by income (<$15,000 and ≥$15,000), education (<high school and ≥high school), and tooth loss (none and any), respectively. Consistent with the methods described in our paper, for each bacterium, individuals were categorized into carriers and noncarriers according to whether they carried the taxon or not. The associations of ancestry with bacterium prevalence were evaluated using logistic regression analyses. Consistent with the stratified analyses for the common taxa, when stratified by one covariate, all other covariates were adjusted in regression models. As shown in Table 2, all four periodontal bacteria showed consistent association with ancestry (*P* < 0.05) in both strata for each variable. These results suggest that the difference of prevalence of these four periodontal bacteria between AAs and EAs was less likely attributable to the differences in income, education, and tooth loss between the two groups.

**TABLE 1:** Associations of ancestry with abundance of 13 common taxa, stratified by age, gender, income, education, smoking, and tooth loss^*a*^

Taxon	Group	Age		Gender		Income		Education		Smoking		Tooth loss	
		Coefficient	*P* value	Coefficient	*P* value	Coefficient	*P* value	Coefficient	*P* value	Coefficient	*P* value	Coefficient	*P* value
Phylum *Actinobacteria*	Overall	−0.44	5.92 × 10^−12^	−0.37	6.38 × 10^−9^	−0.37	2.49 × 10^−9^	−0.44	5.92 × 10^−12^	−0.44	6.88 × 10^−12^	−0.38	1.95 × 10^−9^
	1	−0.32	3.68 × 10^−5^	−0.26	5.26 × 10^−3^	−0.51	6.58 × 10^−7^	−0.58	6.10 × 10^−5^	−0.62	7.35 × 10^−9^	−0.53	8.40 × 10^−8^
	2	−0.48	2.57 × 10^−5^	−0.53	6.05 × 10^−9^	−0.24	2.12 × 10^−3^	−0.40	1.63 × 10^−8^	−0.30	1.43 × 10^−4^	−0.27	1.90 × 10^−3^
Family *Micrococcaceae*	Overall	−0.47	6.58 × 10^−10^	−0.40	1.93 × 10^−7^	−0.39	1.75 × 10^−7^	−0.47	6.58 × 10^−10^	−0.47	8.30 × 10^−10^	−0.41	1.39 × 10^−7^
	1	−0.38	5.81 × 10^−5^	−0.25	0.02	−0.57	3.33 × 10^−6^	−0.51	3.62 × 10^−3^	−0.66	3.49 × 10^−7^	−0.55	3.81 × 10^−6^
	2	−0.48	4.18 × 10^−4^	−0.62	1.90 × 10^−8^	−0.22	0.02	−0.47	3.84 × 10^−8^	−0.35	2.57 × 10^−4^	−0.30	4.04 × 10^−3^
Genus *Rothia*	Overall	−0.49	3.66 × 10^−10^	−0.41	1.28 × 10^−7^	−0.40	1.06 × 10^−7^	−0.49	3.66 × 10^−10^	−0.49	4.54 × 10^−10^	−0.42	1.04 × 10^−7^
	1	−0.40	3.85 × 10^−5^	−0.27	0.02	−0.58	3.92 × 10^−6^	−0.51	3.65 × 10^−3^	−0.67	3.70 × 10^−7^	−0.57	2.54 × 10^−6^
	2	−0.48	4.00 × 10^−4^	−0.63	1.61 × 10^−8^	−0.24	0.01	−0.49	1.93 × 10^−8^	−0.37	1.63 × 10^−4^	−0.30	3.90 × 10^−3^
Species Rothia mucilaginosa	Overall	−0.48	4.97 × 10^−9^	−0.40	1.02 × 10^−6^	−0.38	1.85 × 10^−6^	−0.48	4.97 × 10^−9^	−0.48	6.11 × 10^−9^	−0.40	1.02 × 10^−6^
	1	−0.44	1.55 × 10^−5^	−0.26	0.03	−0.58	8.18 × 10^−6^	−0.50	6.76 × 10^−3^	−0.69	5.40 × 10^−7^	−0.54	2.20 × 10^−5^
	2	−0.35	0.02	−0.63	7.99 × 10^−8^	−0.21	0.04	−0.49	1.07 × 10^−7^	−0.35	7.85 × 10^−4^	−0.30	7.22 × 10^−3^

Phylum *Bacteroidetes*													
Family *Porphyromonadaceae*	Overall	0.45	7.92 × 10^−5^	0.37	1.34 × 10^−3^	0.41	2.25 × 10^−4^	0.45	7.92 × 10^−5^	0.45	7.95 × 10^−5^	0.43	2.25 × 10^−4^
	1	0.20	0.13	0.42	0.02	0.45	0.01	0.54	0.04	0.58	9.67 × 10^−4^	0.53	1.54 × 10^−3^
	2	0.72	2.82 × 10^−3^	0.37	0.02	0.39	8.55 × 10^−3^	0.43	7.31 × 10^−4^	0.38	0.01	0.37	0.02
Genus *Porphyromonas*	Overall	0.44	1.92 × 10^−4^	0.36	2.64 × 10^−3^	0.40	5.24 × 10^−4^	0.44	1.92 × 10^−4^	0.44	1.92 × 10^−4^	0.42	4.67 × 10^−4^
	1	0.18	0.18	0.39	0.03	0.45	0.02	0.51	0.06	0.56	2.33 × 10^−3^	0.50	3.71 × 10^−3^
	2	0.71	4.13 × 10^−3^	0.38	0.02	0.39	0.01	0.43	1.34 × 10^−3^	0.38	0.02	0.38	0.03
Species Prevotella denticola	Overall	0.58	1.68 × 10^−6^	0.47	1.08 × 10^−4^	0.54	5.73 × 10^−6^	0.58	1.68 × 10^−6^	0.58	1.90 × 10^−6^	0.53	1.90 × 10^−5^
	1	0.30	0.04	0.54	4.33 × 10^−3^	0.71	1.41 × 10^−4^	0.88	1.40 × 10^−3^	0.78	6.63 × 10^−5^	0.66	2.31 × 10^−4^
	2	0.93	1.05 × 10^−4^	0.47	5.61 × 10^−3^	0.37	0.02	0.53	1.26 × 10^−4^	0.45	5.30 × 10^−3^	0.40	0.02

Phylum *Firmicutes*													
Family *Carnobacteriaceae*	Overall	−0.23	7.75 × 10^−4^	−0.23	1.08 × 10^−3^	−0.27	9.22 × 10^−5^	−0.23	7.75 × 10^−4^	−0.23	7.78 × 10^−4^	−0.22	1.51 × 10^−3^
	1	−0.28	1.00 × 10^−3^	−0.24	0.02	−0.08	0.43	−0.15	0.32	−0.21	0.05	−0.32	2.28 × 10^−3^
	2	−0.12	0.34	−0.27	6.62 × 10^−3^	−0.30	7.46 × 10^−4^	−0.26	6.96 × 10^−4^	−0.28	1.81 × 10^−3^	−0.15	0.11
Genus *Granulicatella*	Overall	−0.24	5.51 × 10^−4^	−0.24	8.09 × 10^−4^	−0.28	7.09 × 10^−5^	−0.24	5.51 × 10^−4^	−0.24	5.52 × 10^−4^	−0.23	1.28 × 10^−3^
	1	−0.30	6.39 × 10^−4^	−0.26	0.01	−0.09	0.44	−0.16	0.31	−0.22	0.05	−0.33	1.87 × 10^−3^
	2	−0.12	0.34	−0.28	6.20 × 10^−3^	−0.32	5.39 × 10^−4^	−0.27	4.79 × 10^−4^	−0.30	1.30 × 10^−3^	−0.16	0.09
Species Granulicatella adiacens	Overall	−0.25	4.60 × 10^−4^	−0.24	9.07 × 10^−4^	−0.28	8.45 × 10^−5^	−0.25	4.60 × 10^−4^	−0.25	4.60 × 10^−4^	−0.23	1.48 × 10^−3^
	1	−0.31	5.37 × 10^−4^	−0.26	0.01	−0.08	0.48	−0.16	0.32	−0.24	0.04	−0.34	1.81 × 10^−3^
	2	−0.11	0.40	−0.28	6.29 × 10^−3^	−0.32	5.25 × 10^−4^	−0.28	3.58 × 10^−4^	−0.30	1.23 × 10^−3^	−0.16	0.10
Species Streptococcus oligofermentans	Overall	−0.37	4.73 × 10^−5^	−0.42	2.48 × 10^−6^	−0.52	6.61 × 10^−9^	−0.37	4.73 × 10^−5^	−0.37	6.46 × 10^−5^	−0.42	3.71 × 10^−6^
	1	−0.46	2.99 × 10^−5^	−0.66	3.68 × 10^−6^	−0.36	0.01	0.12	0.57	−0.32	0.02	−0.42	2.98 × 10^−3^
	2	−0.38	0.02	−0.23	0.06	−0.51	1.64 × 10^−5^	−0.48	2.33 × 10^−6^	−0.41	7.70 × 10^−4^	−0.38	2.05 × 10^−3^
Species *Streptococcus* sp. oral taxon 057	Overall	−0.22	3.84 × 10^−5^	−0.17	1.07 × 10^−3^	−0.18	4.29 × 10^−4^	−0.22	3.84 × 10^−5^	−0.22	4.02 × 10^−5^	−0.18	1.02 × 10^−3^
	1	−0.14	0.04	−0.14	0.08	−0.24	3.63 × 10^−3^	−0.27	0.02	−0.38	2.67 × 10^−5^	−0.27	1.32 × 10^−3^
	2	−0.25	6.56 × 10^−3^	−0.24	1.36 × 10^−3^	−0.10	0.14	−0.22	2.30 × 10^−4^	−0.09	0.19	−0.10	0.16
Family *Peptostreptococcaceae*	Overall	0.45	8.46 × 10^−7^	0.38	2.93 × 10^−5^	0.42	2.75 × 10^−6^	0.45	8.46 × 10^−7^	0.45	8.43 × 10^−7^	0.43	4.94 × 10^−6^
	1	0.25	0.02	0.39	5.61 × 10^−3^	0.61	2.69 × 10^−5^	0.68	1.26 × 10^−3^	0.63	2.89 × 10^−5^	0.66	2.16 × 10^−6^
	2	0.74	2.29 × 10^−5^	0.42	8.56 × 10^−4^	0.26	0.03	0.38	1.88 × 10^−4^	0.36	2.55 × 10^−3^	0.30	0.02

a For each sample, centered log-ratio transformation was used to normalize taxon read counts. The associations of taxon abundance with ancestry were evaluated using linear regression analyses.

**TABLE 2:** Associations of ancestry with abundance of four periodontal bacteria, stratified by income, education, and tooth loss^*a*^

Oral pathogen	Group	Income		Education		Tooth loss	
		Coefficient	*P* value	Coefficient	*P* value	Coefficient	*P* value
Porphyromonas gingivalis	Overall	0.85	4.53 × 10^−10^	0.75	7.37 × 10^−8^	0.77	2.91 × 10^−8^
	1	0.61	6.39 × 10^−3^	1.25	1.28 × 10^−4^	0.89	5.72 × 10^−5^
	2	0.94	3.68 × 10^−7^	0.65	4.47 × 10^−5^	0.63	7.37 × 10^−4^

Prevotella intermedia	Overall	0.87	1.73 × 10^−9^	0.79	6.30 × 10^−8^	0.82	1.43 × 10^−8^
	1	1.05	5.81 × 10^−6^	1.36	5.56 × 10^−5^	0.89	1.56 × 10^−4^
	2	0.66	6.46 × 10^−4^	0.63	1.50 × 10^−4^	0.74	1.14 × 10^−4^

Filifactor alocis	Overall	0.78	3.35 × 10^−9^	0.67	6.32 × 10^−7^	0.70	1.20 × 10^−7^
	1	0.68	1.14 × 10^−3^	1.01	1.10 × 10^−3^	0.86	2.81 × 10^−5^
	2	0.80	9.35 × 10^−6^	0.62	5.92 × 10^−5^	0.60	1.15 × 10^−3^

Treponema denticola	Overall	0.65	1.09 × 10^−6^	0.59	1.52 × 10^−5^	0.61	4.19 × 10^−6^
	1	0.83	9.52 × 10^−5^	1.04	8.24 × 10^−4^	0.80	1.07 × 10^−4^
	2	0.41	0.02	0.53	7.35 × 10^−4^	0.47	9.88 × 10^−3^

a For each taxon, individuals were categorized into carriers and noncarriers according to whether they carried the taxon or not. The associations of taxon prevalence with ancestry were evaluated using logistic regression analyses.

We applaud Dr. Kenyon for pondering the potential inadequate adjustment of covariates and raising the possibility that the different prevalence of the four periodontal bacteria between the two groups may be due to differences in oral health and socioeconomic status. However, as elucidated by analyses, these results are unlikely to be due to these potential biases.
